# Flavonoids of *Tetrastigma hemsleyanum* Diels et Gilg Against Acute Hepatic Injury by Blocking PI3K/AKT Signaling Pathway

**DOI:** 10.1155/mi/4302130

**Published:** 2025-01-07

**Authors:** Lingling Li, Lianghui Zhan, Xiaojun Wu, Xuechun Jiang, Jinbao Pu

**Affiliations:** ^1^School of Pharmaceutical Sciences, Zhejiang Chinese Medical University, Hangzhou 310053, China; ^2^Tongde Hospital of Zhejiang Province, Hangzhou 310014, China; ^3^Center for Medicinal Resources Research, Zhejiang Academy of Traditional Chinese Medicine, Hangzhou 310007, China; ^4^Zhejiang Provincial Key Laboratory of Traditional Chinese Medicine New Drug Research and Development, Zhejiang, China

**Keywords:** acute hepatic injury, flavonoids, molecular dock, network pharmacology, *Tetrastigma hemsleyanum* Diels et Gilg

## Abstract

**Objective:** This study aims to investigate the mechanism of *Tetrastigma hemsleyanum* Diels et Gilg flavonoids (THF) on acute hepatic injury (AHI).

**Methods:** First, high-performance liquid chromatography (HPLC) fingerprints were established to obtain the main chemical components of THF. According to the network pharmacology databases, collect active targets of AHI and potential targets. Using interaction targets to construct a protein–protein interaction (PPI) network, followed by Gene Ontology (GO) and Kyoto Encyclopedia of Genes and Genomes (KEGG) enrichment analysis. Finally, the affinity between the core targets and the main active ingredients was verified by molecular docking. Next, verified network pharmacology predictions with animal experiments. Mice were treated with THF (20, 40, and 80 mg/kg) for 7 days, and then built an acute liver injury model (lipopolysaccharide [LPS], 10 mg/kg). Detecting the liver biochemical indices, observe the liver pathological changes, and verify the key signaling pathway targets.

**Results:** HPLC showed that the main components of THF were quercetin and kaempferol. Seven active ingredients and 193 potential targets were screened, and 259 disease targets related to acute liver injury, quercetin, and kaempferol may be the main active ingredients in THF. PPI network analysis showed that tumor necrosis factor (TNF), interleukin-6 (IL-6), and tumor protein 53 (TP53) were potential targets of THF for the treatment of AHI. KEGG analysis showed that the phosphoinositide 3-kinase (PI3K)/protein kinase B (AKT) signaling pathway might be one of the main pathways in the treatment of AHI. The molecular docking results showed that active compounds both have strong binding activity with potential targets in PPI. In vivo experiments showed that THF could reduce the fibrosis and inflammation of liver tissue etc. Meanwhile, it could downregulate the alanine aminotransferase (ALT), aspartate aminotransferase (AST), IL-6, tumor necrosis factor alpha (TNF-α), C-reactive protein (CRP) levels, and the protein expressions of phosphorylated phosphoinositide 3-kinase (p-PI3K), phosphorylated protein kinase B (p-AKT), and the ratio of BCL2-associated X (BAX)/B-cell lymphoma-2 (BCL-2) in the liver tissue of the mice with acute liver injury and upregulate the level of interleukin-10 (IL-10).

**Conclusion:** The treatment of acute liver injury with THF is characterized by multicomponents and multitargets, and its mechanism may be related to the alleviation of the inflammatory response, reduction of apoptosis, and regulation of the PI3K/AKT signaling pathway.

## 1. Introduction

The liver, being the principal organ responsible for detoxification, exhibits remarkable resistance against bacterial and viral infections as well as drug-induced toxicity [1]. Acute hepatic injury (AHI) is a highly prevalent and fatal condition among liver diseases, primarily attributed to the inappropriate use of medications [2]. The pathogenic mechanisms underlying lipopolysaccharide (LPS)-induced AHI resemble those triggered by hepatotoxic drugs such as acetaminophen [3]. In response to LPS stimulation, hepatic cells recruit immune cells to orchestrate the production of proinflammatory cytokines, thereby initiating an inflammatory cascade and leading to tissue damage [4]. The current hepatoprotective drugs, such as silymarin, exhibit limited therapeutic efficacy and are associated with a multitude of adverse effects, indicating the need to develop more effective and safer drugs for AHI treatment [5, 6].

Traditional Chinese medicine (TCM) is widely used in China due to its advantages of multiplying metabolites with several targets and fewer side effects. *Tetrastigma hemsleyanum* Diels et Gilg (TH), being a valuable botanical resource in our country, boasts a rich history spanning thousands of years within traditional medicine [7]. Previous studies have demonstrated that flavonoids, as the bioactive compounds found in TH, exhibit significant therapeutic efficacy against AHI [8, 9]. However, the pharmacological substance basis and potential mechanism of action for the treatment of AHI with TH flavonoids (THF) are unclear and require further exploration.

This research employed network pharmacology to predict the active compounds and potential targets as well as pathways of THF in the treatment of AHI. Next, the network pharmacology results were confirmed using molecular docking. Finally, validated the projected targets and mechanisms of action via in vivo experiments. The present study serves as the theoretical basis for the therapeutic use of TH in AHI and offers novel sight of AHI treatment.

## 2. Materials and Methods

### 2.1. Network Pharmacology Analysis

#### 2.1.1. Active Ingredients and Targets of THF Acquisition

We collect the THF active ingredients by searching the Chinese National Knowledge Infrastructure (CNKI, https://www.cnki.net) and PubMed databases, and their corresponding targets were searched in the Traditional Chinese Medicine Systems Pharmacology (TCMSP) database and Analysis Platform database (http://tcmspw.com/tcmsp.php). Next, those compounds were screened based on oral bioavailability (OB) ≥30% and drug-likeness (DL) ≥0.18, collected the obtained active ingredients, were then summarized, and integrated the active targets of each component. THF active ingredients were converted into gene names by the UniProt database (http://www.uniprot.org/).

#### 2.1.2. Targets of AHI Collection

We use “acute hepatic injury” or “acute liver injury” as the keywords to get the targets from the GeneCards database (https://auth.lifemapsc.com/), the Online Mendelian Inheritance in Man (OMIM) database (https://www.omim.org/), and Therapeutic Target Database (TTD) database (http://db.idrblab.net/ttd/). And those putative targets species were limited as “*Homo sapiens*.” Then we eliminate the repeated value in order to determine the related putative value. We transform these targets into gene names in the UniProt database (http://www.uniprot.org/).

In addition, intersection targets were obtained by Bioinformatics (http://www.bioinformatics.com.cn/), analyzing component targets and disease targets and additionally identifying the possible treatment targets of THF for AHI. At last, we use these targets to draw a Veen map.

#### 2.1.3. “THF–Compound–AHI–Target” Construction

Veen map in Section 2.1.2 showed the intersection targets. In addition, herbs, active compounds, intersection targets, and diseases were imported into Cytoscape 3.6.1. The network of “THF–compound–AHI–target” was established based on the correspondence and attributes.

#### 2.1.4. Constructing the Protein–Protein Interaction (PPI) Network and Core Target Screening

We uploaded the intersection targets in Section 2.1.2. to the Search Tool for the Retrieval of Interaction Gene/Proteins (STRING) database (https://string-db.org/) to build the PPI network. Besides, the species were limited to being “*Homo sapien*,” with a medium confidence level established as “0.4.” We imported the obtained PPI network to Cytoscape 3.6.1 for visual analysis.

#### 2.1.5. Gene Ontology (GO) Annotation and Kyoto Encyclopedia of Genes and Genomes (KEGG) Enrichment Analysis

The key genes were imported into the DAVID database (https://david.ncifcrf.gov/) to get GO and KEGG analysis data, with the screening conditions set at *p* < 0.05. Following this, data from GO and KEGG were uploaded to the Bioinformatics platform (http://www.bioinformatics.com.cn/) to get a visual analysis.

#### 2.1.6. Molecular Docking Analysis

Search for the target proteins tumor necrosis factor (TNF), interleukin-6 (IL-6), and TP53 in the RCSB PDB (http://www.rcsb.org/pdb/home/home.do) (with the restrictions that the source organism is *Homo sapiens*, the experimental method is X-ray diffraction, and the resolution is ≤3). Target proteins with small molecule ligands were preferred as receptors and saved as PDB format files. Quercetin and kaempferol, the main components of THF, were downloaded from the PubChem database (https://pubchem.ncbi.nlm.nih.gov/) and saved as SDF format files to be used as ligands. After that, the ligand small molecules (quercetin and kaempferol) and target proteins (TNF, IL-6, and TP53) were imported into Discovery Studio 4.5 Client. Redundant conformations and water molecules of the proteins were removed, and hydrogen additions, charge calculations, and receptor assignments were performed. Binding sites of protein pockets were predicted and removed primitive ligands. Molecular docking was then performed using the main components as ligands for target proteins, respectively, exported as 3D and 2D plots. The binding stability between ligand and receptor was negatively correlated with the binding energy, with binding energies below −5.0 kcal/mol indicating effective interactions, while those below −7.0 kcal/mol indicated strong binding activity.

### 2.2. Animal Experiment

#### 2.2.1. Preparation of THF

An amount of TH slices was weighed and soaked in 60% ethanol for 30 min at a 1:10 ratio. Next, we extracted it with 60% ethanol at a constant temperature of 90°C for 2 h and repeated it two times. The two filtrates were combined and concentrated to a certain concentration (3.4−5 mg/mL). Firstly, the HPD 826 macroporous resin was soaked in anhydrous ethanol for 24 h and fully activated. 2BV water was washed until there was no smell of ethanol and then washed with 4% hydrochloric acid and then with 4% sodium hydroxide. After washing to neutral water, the samples were loaded. Next, we have the following: sample volume:column volume = 1:1, flow rate, 2 BV/h, and 2 BV water elution followed by 3 BV 70% ethanol, flow rate 2 BV/h, collected the filtrate [10]. After concentration and drying the filtrate, the purity of the powder was 70% (shown in the Suppoting Information 1: Table S1).

#### 2.2.2. THF Component Analysis by High-Performance Liquid Chromatography (HPLC)

The HPLC assay conditions were as follows, Agilent 1260 infinity II HPLC-DAD system, Agilent XDB C-18 (5 μm, 12 nm, 4.6 mm × 250 mm); mobile phase, 0.085% phosphoric acid (A)-acetonitrile (B); gradient of elution, 0–15 min 10% B, 15–30 min 35% B, 30–55 min 100% B, 55–60 min 100% B; post-time, 8 min; and detect wavelength at 372 nm.

#### 2.2.3. Animal

Male ICR mice (19–21 g) were provided by Hangzhou Medical College (License No. SCXK (Zhe) 2019-0002, SPF). Mice were housed in individual mouse cages at the temperature with 18−22°C (40%–60% relative humidity) when there was normal lighting and unrestricted access to water. These procedures were approved by the Ethics Committee for the Zhejiang Academy of Traditional Chinese Medicine (Hangzhou, China).

#### 2.2.4. Method of Administration and LPS-Induced AHI Model

All mice were randomly into five groups (10 mice in every group): control (CON, given the same volume of water as THF groups), model (MOD, given the same volume of water as THF groups), THF of high dose (THF-H, 80 mg/kg THF), medium dose (THF-M, 40 mg/kg THF), and low dose (THF-L, 20 mg/kg THF) group [11]. THF group mice were given THF for 7 days. AHI models were induced by intraperitoneal injection of LPS (10 mg/kg), and the CON group was given water as the same volume of MOD group [12]. On the seventh day, collect the blood and liver tissues for the next research. Liver tissues underwent a rinse with cold saline and were split into two segments, with the initial segment being preserved using 10% neutral formaldehyde for histopathological analysis, and the latter segment was mixed into a homogenate to identify protein expression.

#### 2.2.5. The Determination of Alanine Aminotransferase (ALT), Aspartate Aminotransferase (AST), and Inflammatory Factor Levels

Following a 10-min centrifugation process at a rate of 3500 r/min, the levels of serum alanine ALT and AST were determined through liver function testing kits, following the guidelines provided by the manufacturer. The levels of IL-6, interleukin-10 (IL-10), tumor necrosis factor alpha (TNF-α), and C-reactive protein (CRP) in the liver tissues were quantified using biochemical and ELISA kits, adhering to the manufacturer's guidelines.

#### 2.2.6. Hepatic Histopathology of AHI

Initially, the liver samples were preserved in 10% neutral formaldehyde for 24 h at room temperature, embedded in paraffin, and cut into 4-µm-thick slices. After complete dewaxing, one section was stained with hematoxylin and eosin (H&E), and the other section was used for Masson staining.

Images capturing the H&E staining livers were taken using an optical microscope at a magnification of 200×, while the Masson staining images were taken at 400× magnification.

#### 2.2.7. Immunohistochemical (IHC) Detection of Phosphorylated Phosphoinositide 3-Kinase (p-PI3K) and Phosphorylated Protein Kinase B (p-AKT) in AHI

The pathology sections were completely dewaxed and washed three times in phosphate buffer solution (PBS) for 5 min each time. Mext, antigen repair was performed with antigen repair solution microwave on high for 8 min. After cooling to room temperature, the sections were quenched with 3% hydrogen peroxide. The sections were washed three times in PBS for 5 min each time. Subsequently, the sections were blocked with 5% bovine serum albumin (BSA) for 2 h. Sections were incubated with primary antibodies (1:200 for p-AKT, 1:200 for p-PI3K) at 4°C overnight. The next day, after rewarming, the sections were rinsed with PBS and incubated with secondary antibody (HRP-IgG 1:8000) for 20 min at 37°C. The sections were stained with DAB for 5 min, stained with hematoxylin, differentiated, dehydrated, cleared, and mounted, and then IHC results were obtained. IHC results of p-PI3K and p-AKT in the liver were analyzed using ImageJ.

#### 2.2.8. TUNEL Detection of Apoptosis in the Liver of AHI Mice

After the slices were completely dewaxed, distilled water was used for 2 min. Twenty micrograms per milliliter proteinase K was incubated at 37°C for 20 min and rinsed in PBS three times, each time for 5 min, and TUNEL detection solution was added dropwise (TdT Enzyme:FITC-12-dUTP labeling mix of 1:9), and the reaction was closed to light at 37°C for 60 min and rinsed in PBS three times, each time for 5 min. The reaction was carried out at 37°C for 60 min, rinsed with PBS for 3 times, each time for 5 min, and sealed with antifluorescence quencher, and the TUNEL-positive area showed red fluorescence when observed under fluorescence microscope. Analysis of apoptosis results in liver using ImageJ.

#### 2.2.9. Western Blotting Analysis

Weigh approximately 80 mg of liver tissue, wash off the blood, add 400 μL of RIPA lysis buffer containing PMSF and grind well, extract the total protein in the liver tissue, and utilize the kit to ascertain the concentration of protein. Subsequently, the protein underwent separation using either 10% or 15% SDS–PAGE on the PVGF membrane. The membranes were blocked in 5% nonfat milk for 2 h at room temperature before incubation with antibodies against AKT1 (1:1500), PI3K (1:1000), p-AKT (1:1000), p-PI3K (1:1000), BAX (1:6000), BCL-2(1:1500), and β-actin (1:8000) at 4°C overnight. Each membrane underwent three 10-min washes using TBST, followed by a 2-h room temperature incubation with HRP-IgG (1:8000). At last, chemiluminescence (ECL) was employed to make the membranes visible. Use ImageJ to analyze protein grayscale values.

### 2.3. Statistical Analysis

All experimental data were statistically analyzed using IBM SPSS 22.0 software (SPSS Inc., NY, USA). The results were expressed as mean ± standard deviation (SD) ($\overset{―}{x}$ ± s), and one-way analysis of variance (ANOVA) was used for comparison among groups. A *p* value below 0.05 was deemed to hold statistical significance.

## 3. Results

### 3.1. Detection and Quantification of THF

**Figure 1:**
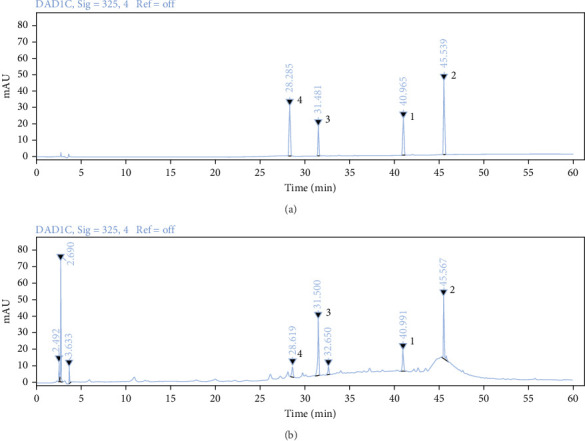
HPLC of the standard and THF sample at 372 nm: (A) standard diagram and (B) sample diagram. 1, kaempferol; 2, quercetin; 3, nicotifiorin; 4, vitexin.

Use HPLC analysis to identify the THF (Figure 1). According to the methods, the standard curves of quercetin were *y* = 0.0164*x* − 0.0963 (*R*^2^ = 0.9989), the standard curves of kaempferol were *y* = 0.0173*x* − 0.21 (*R*^2^ = 0.9985), the standard curves of nicotifiorin were *y* = 0.0578*x* − 0.3037 (*R*^2^ = 0.9985), and the standard curves of vitexin were *y* = 0.0528*x* − 0.3772 (*R*^2^ = 0.9985). Based on the test results, it can be concluded that THF had four main compounds; they are quercetin (90.88 ± 0.004 μg/g), kaempferol (18.85 ± 0.008 μg/g), nicotifiorin (209.37 ± 0.007 μg/g), and vitexin (108.74 ± 0.004 μg/g).

### 3.2. Network Pharmacology Research

#### 3.2.1. Active Ingredients of THF

The THF collected from the TCMSP database contained seven main active ingredients (Table 1), which corresponded to 193 targets, with the qualification lists of “OB ≥ 30%” and “DL ≥ 0.18”. As seen in Table 1, the highest number of targets of the 7 THF active ingredients was quercetin with 74 targets, followed by kaempferol (43 targets), and isorhamnetin (31 targets).

#### 3.2.2. Intersection Targets of THF and AHI

**Figure 2:**
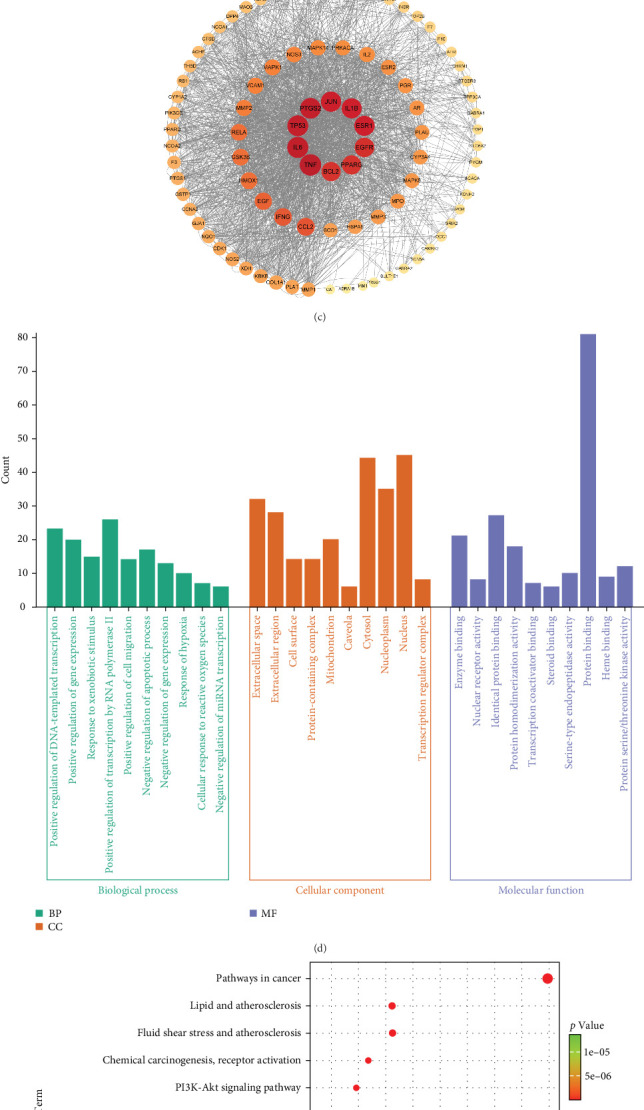
Network pharmacology figures. (A) Venn diagram of common targets of THF and AHI. (B) THF–compound–AHI–target network. (Active THF names are marked by green squares, and the yellow circles indicate the THF treatment of AHI-related common targets.) (C) PPI network of related THF targets in the treatment of AHI. (D) GO enrichment analysis. (E) Enrichment analysis of KEGG pathways. AHI, acute hepatic injury; GO, Gene Ontology; KEGG, Kyoto Encyclopedia of Genes and Genomes; PPI, protein–protein interaction; THF, *Tetrastigma hemsleyanum* Diels et Gilg flavonoids.

AHI had 2806 targets from the OMIM, Gene Cards, and TTD databases. Obviously, the Venn map revealed that the integration of AHI targets with THF targets resulted in 91 intersecting targets (deleting duplicate values), which can be used as the primary objectives for the treatment of AHI (Figure 2A).

#### 3.2.3. Construction of the THF–Compound–AHI–Target Network

Through the Cytoscape software, a network graph comprising 91 nodes and 1015 edges was developed (Figure 2B). The network included 7 flavonoid active components of THF, 193 THF targets, 1 disease, and 91 potential therapeutic targets. As can be seen, the application of THF to AHI may target various elements across different compounds, illustrating the multicomponent and multitarget of THF's AHI treatment.

#### 3.2.4. PPI Network Analysis

To search the role of the relationship between THF and AHI intersecting genes, the PPI network was developed by importing the overlapping genes of THF and AHI into the STRING database, with 91 nodes and 1015 connecting lines (Figure 2C). Among them, the degree of TNF, IL6, TP53, PTGS2, JUN, ESR1, and IL1B were all greater than 110, and these targets were mainly involved in inflammatory response, apoptosis, and gene expression; among them, the TNF degree was the highest (degree = 124.0). Drugs could improve acute liver injury by inhibiting the levels of inflammatory and apoptotic proteins such as TNF, TP53, and IL6. These suggested that TNF, IL6, and TP53 might be the main targets for THF treatment of AHI.

#### 3.2.5. GO Functional Enrichment of Targets Associated With THF for AHI Treatment

Through the GO function enrichment analysis, 406 biological processes (BPs), 55 cellular components (CCs), and 406 molecular functions (MFs) were obtained, which accounted for 46.83%, 6.34%, and 46.83%. See the histogram with top 10 entries (*p* < 0.05) (Figure 2D). Among them, BP was mainly involved in the positive regulation of gene expression, positive regulation of gene expression, and response to xenobiotic stimulus, suggesting that THF could be used as an effective mechanism to regulate gene expression. CC enrichment mainly involves the plasma membrane and other CCs of secretory cells; MF enrichment mainly involves protein binding and enzyme binding.

#### 3.2.6. Enrichment Analysis of KEGG Pathway for THF Treatment of AHI-Related Targets

Analysis of the KEGG pathways revealed the involvement of 155 KEGG pathways in the objectives linked to THF therapy for AHI, and the top 10 pathways were selected according to *p* < 0.05, as shown in Figure 2E. KEGG enrichment bubble plots showed that the PI3K-AKT, IL-17, and chemical carcinogenesis–reactive oxygen species signaling pathways had the correlation with many BPs, including inflammation, cell proliferation, and cell apoptosis among those pathways. This suggested that THF cured AHI through PI3K–AKT and some inflammation pathways.

#### 3.2.7. Molecular Docking Analysis

To further evaluate the impact of THF on AHI, molecular docking including quercetin and kaempferol with core target proteins (TNF, IL6, and TP53) was conducted. TNF (PDB ID: 7jra), IL-6 (PDB ID: 1alu), and TP53 (PDB ID: 8dc6) were selected as suitable protein structures for those genes, respectively.

**Figure 3:**
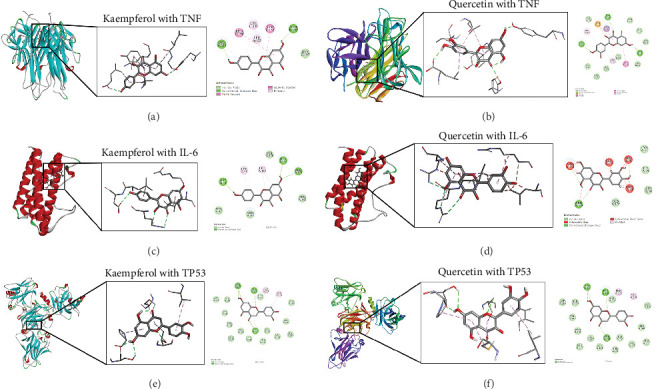
The protein–ligand of the docking simulation (3D and 2D figures). (A) Kaempferol with TNF. (B) Quercetin with TNF. (C) Kaempferol with IL-6. (D) Quercetin with IL-6. (E) Kaempferol with TP53. (F) Quercetin with TP53. IL-6, interleukin-6; TNF, tumor necrosis factor.

The study results show that the binding energies of these components with the core targets are all <−5.0 kcal/mol, showing that all of these targets bind effectively to the compounds. The binding energy of kaempferol with TNF is −8.07 kcal/mol, the quercetin with TNF is −7.51 kcal/mol, and the kaempferol with TP53 is −7.01 kcal/mol (Table 2); these showed the strong binding forces through hydrogen bonding. And docking 3D and 2D figures were shown in Figure 3.

### 3.3. Animal Experimental Research

#### 3.3.1. THF Ameliorates Pathologic Damage in AHI

In order to determine the severity of liver injury in mice, H&E staining was used to examine the pathological changes in the liver of mice and Masson staining to examine the degree of hepatic fibrosis in mice.

**Figure 4:**
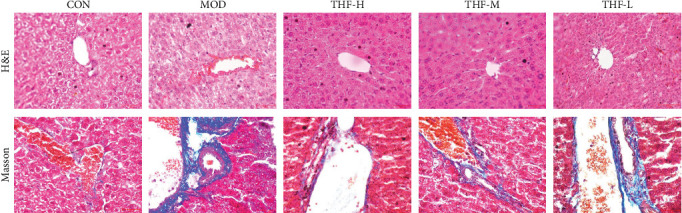
Histopathological examination of liver tissue (HE staining, 200×; Masson staining 400×). CON, control; H&E, hematoxylin and eosin; MOD, model; THF-H, *Tetrastigma hemsleyanum* Diels et Gilg flavonoids of high dose; THF-L, *Tetrastigma hemsleyanum* Diels et Gilg flavonoids of low dose; THF-M, *Tetrastigma hemsleyanum* Diels et Gilg flavonoids of medium dose.

As shown in Figure 4, the CON group had normal liver tissue structure, uniform and orderly cell size, no inflammatory cell infiltration, and no hepatocyte necrosis. The liver lobule of the MOD group mice showed obvious damage, manifested as hepatocyte swelling, vacuolation of cytoplasm, and inflammatory cell infiltration. Compared with the model group, the liver lobule structure of the THF low, medium, and high dose groups was clearer, the hepatic cord arranged in a radial pattern, and the area of liver tissue necrosis and inflammatory infiltration was significantly reduced.

Masson staining showed that collagen fibers were blue; myofibrils, cytoplasm, and erythrocytes were stained red; and the nucleus of the cells showed blue. The liver tissues of mice in the CON group had less staining of collagen fibers and uniform distribution of myofibers. In the MOD group, there was a significant increase in collagen fibers in the field of view, and the tissue fibrosis was obvious. Compared with the MOD group, the collagen fibers in the liver tissues of mice in the THF groups were gradually reduced, and the fibrosis was effectively alleviated, as shown in Figure 4.

#### 3.3.2. Effect of THF on Liver Function Indices

**Figure 5:**
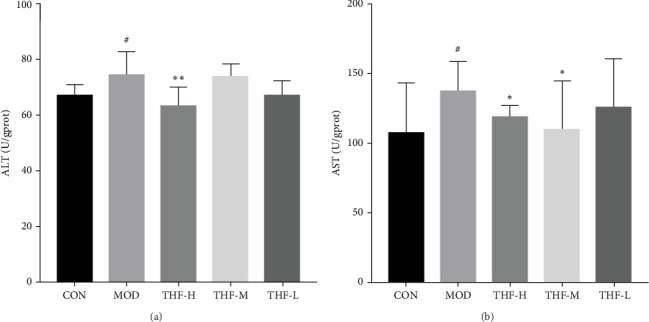
Effect of THF on ALT and AST levels in the liver of AHI. Data are presented as the mean ± standard deviation (SD), *n* = 10. ^#^*p* < 0.05 compared with the CON group; *⁣*^*∗*^*p* < 0.05, and *⁣*^*∗∗*^*p* < 0.01 compared with the MOD group. AHI, acute hepatic injury; ALT, alanine aminotransferase; AST, aspartate aminotransferase; CON, control; MOD, model; THF-H, *Tetrastigma hemsleyanum* Diels et Gilg flavonoids of high dose; THF-L, *Tetrastigma hemsleyanum* Diels et Gilg flavonoids of low dose; THF-M, *Tetrastigma hemsleyanum* Diels et Gilg flavonoids of medium dose.

The levels of AST and ALT biochemical indicators would change when AHI happened. As shown in Figure 5, that LPS significantly increased the secretion of AST and ALT in the MOD group when compared with the CON group (*p* < 0.05, Figure 5A,B). After THF intervention, the mice liver serum the levels of AST and ALT had a noticeable reduction in the THF-H group (Figure 5A,B, *p* < 0.05, 0.01), and the THF-M group also significantly reduced the level of AST (Figure 5B, *p* < 0.05). These suggested that THF has a therapeutic effect on AHI.

#### 3.3.3. Levels of Inflammatory Factors in Mouse Liver Tissue

**Figure 6:**
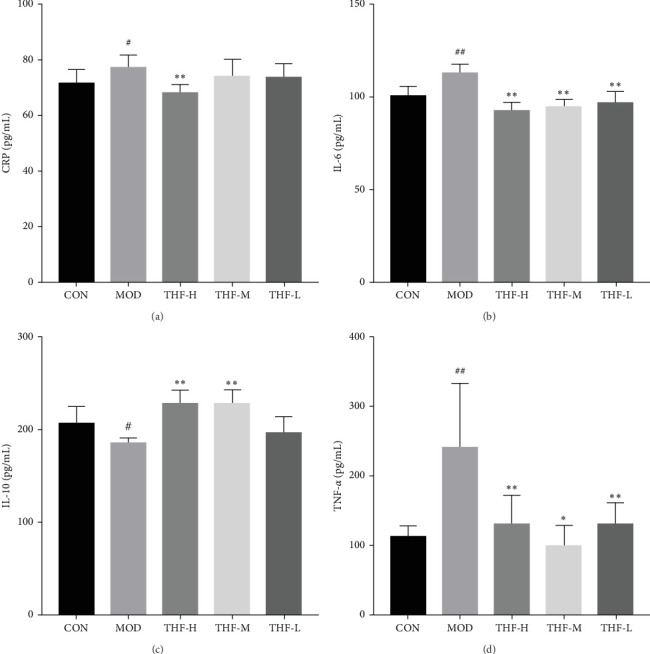
Effect of THF on TNF-α, IL-6, IL-10, and CRP in serum of mice with LPS-induced AHI. And the production of IL-6, IL-10, TNF-α, and CRP was detected by ELISA kits. Data are presented as the mean ± standard deviation (SD), *n* = 10. ^#^*p* < 0.05, ^##^*p* < 0.01 compared with the CON group; *⁣*^*∗*^*p* < 0.05, *⁣*^*∗∗*^*p* < 0.01 compared with the MOD group. AHI, acute hepatic injury; CON, control; CRP, C-reactive protein; IL-6, interleukin-6; IL-10, interleukin-10; LPS, lipopolysaccharide; MOD, model; THF-H, *Tetrastigma hemsleyanum* Diels et Gilg flavonoids of high dose; THF-L, *Tetrastigma hemsleyanum* Diels et Gilg flavonoids of low dose; THF-M, *Tetrastigma hemsleyanum* Diels et Gilg flavonoids of medium dose.

According to PPI network analysis, the targets of THF for AHI are closely related to inflammatory factors (TNF and IL-6). Compared with the CON group, the MOD group had increased levels of CRP, IL-6, and TNF-α and decreased levels of IL-10 (Figure 6A,C, *p* < 0.05; Figure 6B,D, *p* < 0.01). When compared with the MOD group, the THF-H group showed highly significant decreases in CRP, IL6, and TNF-α levels and highly significant increase in IL-10 levels (Figure 6A–D, *p* < 0.01); in the THF-M group, the level of IL-10 was highly significantly increased (Figure 6C, *p* < 0.01), and the level of TNF-*α* was significantly decreased (Figure 6D, *p* < 0.05); the levels of IL-6 and TNF-α inflammatory factors were extremely significantly decreased in the THF-L group (Figure 6B,D, *p* < 0.01). All the above might suggest that THF can reduce the inflammatory response produced by AHI.

#### 3.3.4. Impact of THF on the PI3K/AKT Signaling Pathway

**Figure 7:**
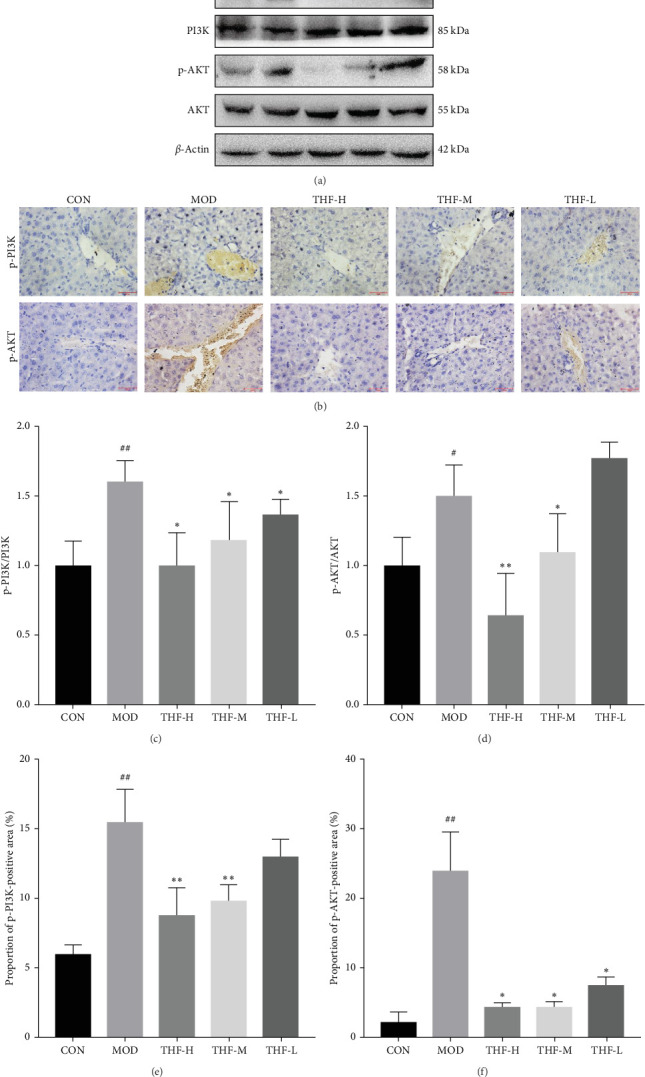
Effect of THF on hepatic PI3K/AKT pathway in AHI mice (400×). Data are presented as the mean ± standard deviation (SD), *n* = 3. ^#^*p* < 0.05, ^##^*p* < 0.01 compared with the CON group; *⁣*^*∗*^*p* < 0.05, *⁣*^*∗∗*^*p* < 0.01 compared with the MOD group. AHI, acute hepatic injury; AKT, protein kinase B; CON, control; MOD, model; p-AKT, phosphorylated protein kinase B; PI3K, phosphoinositide 3-kinase; p-PI3K, phosphorylated phosphoinositide 3-kinase; THF-H, *Tetrastigma hemsleyanum* Diels et Gilg flavonoids of high dose; THF-L, *Tetrastigma hemsleyanum* Diels et Gilg flavonoids of low dose; THF-M, *Tetrastigma hemsleyanum* Diels et Gilg flavonoids of medium dose.

The KEGG pathway suggested that the PI3K/AKT pathway is a relevant pathway for the treatment of AHI. Compared with the CON group, p-PI3K and p-AKT levels were significantly higher in the MOD group (Figure 7C,D, *p* < 0.01, *p* < 0.05). As compared with the model group, p-PI3K, and p-AKT, protein levels were significantly reduced in the THF-H group (Figure 7C, *p* < 0.05; Figure 7D, *p* < 0.01). In the THF-M group, a significant reduction of the p-PI3K and p-AKT protein levels were significantly reduced (Figure 7C, D, *p* < 0.05). In the THF-L group, the level of p-PI3K protein expression was reduced (Figure 7C, *p* < 0.05)

The role of THF on the PI3K/AKT signaling pathway was further determined by IHC. As shown in the results (Figure 7B,E,F), compared with the CON group, the MOD group showed obvious brownish–yellow positive p-PI3K and p-AKT expression; compared with the MOD group, the area of positive expression was significantly reduced in the THF groups (Figure 7B,E,F). The results suggested that THF might exert hepatoprotective effects on AHI mice by inhibiting the PI3K/AKT signaling pathway.

#### 3.3.5. Effect of THF on Apoptotic Factors in AHI Mice

**Figure 8:**
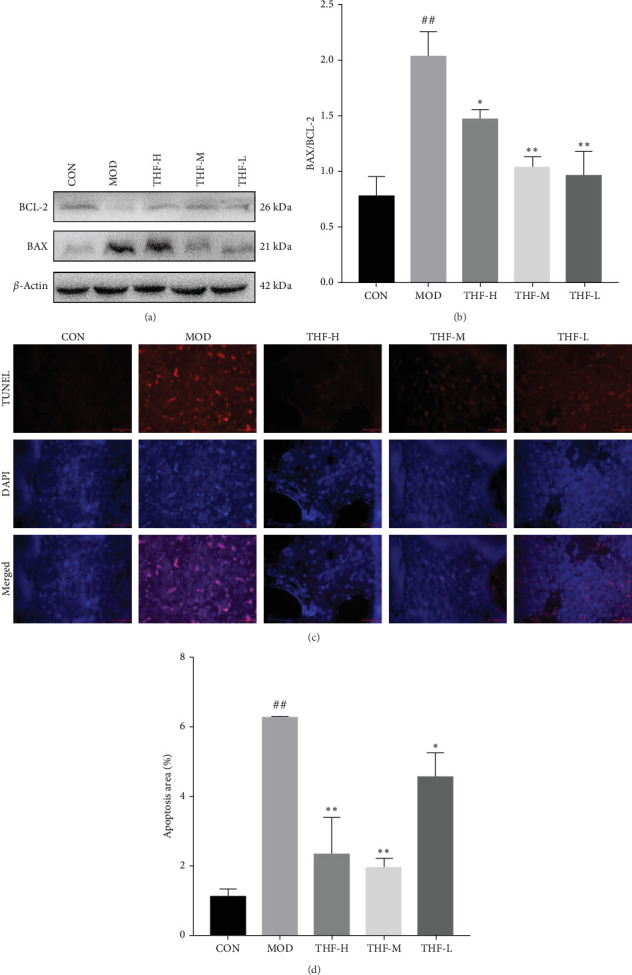
Effect of THF on liver apoptotic proteins BAX and BCL-2 in AHI mice. Data are presented as the mean ± standard deviation (SD), *n* = 3. ^##^*p* < 0.01 compared with the CON group; *⁣*^*∗*^*p* < 0.05, *⁣*^*∗∗*^*p* < 0.01 compared with the MOD group. AHI, acute hepatic injury; BAX, BCL2-associated X; BCL-2, B-cell lymphoma-2; CON, control; MOD, model; THF-H, *Tetrastigma hemsleyanum* Diels et Gilg flavonoids of high dose; THF-L, *Tetrastigma hemsleyanum* Diels et Gilg flavonoids of low dose; THF-M, *Tetrastigma hemsleyanum* Diels et Gilg flavonoids of medium dose.

When the PI3K/AKT signaling pathway was activated, apoptosis was increased. WB results showed that BAX/BCL-2 expression was increased in the MOD group when compared with the CON group (Figure 8A,B, *p* < 0.01). With the increase of THF administration dose, BAX/BCL-2 expression was gradually decreased (Figure 8A,B, *p* < 0.01). This indicated that when AHI occurred, the mice produced cellular apoptosis. Further TUNEL staining showed that the area of positive red fluorescence in the livers of mice in the MOD group was elevated compared with that in the CON group (Figure 8C,D, *p* < 0.01), whereas the red fluorescence expression was significantly reduced in the THF low-dose, medium-dose, and high-dose groups compared with that in the MOD group (Figure 8C,D, *p* < 0.01, *p* < 0.05). Results showed that THF could alleviate the apoptosis induced by LPS to AHI

## 4. Discussion

This study first analyzed the targets of THF treated on AHI disease through network pharmacology and molecular docking and then verified the protective effect of THF on LPS-induced AHI through in vivo experiments. Quercetin and kaempferol were identified as the key targets in treating AHI according to the network pharmacology analysis. Additionally, HPLC analyses revealed that quercetin and kaempferol were the primary constituents of THF. Also in previous studies, quercetin has been found to treat AHI [13]. In some in vivo studies for the treatment of AHI, quercetin and kaempferol significantly reduced hepatocyte disorders and necrosis, which in turn increased the levels of AST and ALT, and decreased the indicators of liver injury [14, 15]. All of these are consistent with the results of the present experiment; pathological results showed that THF could diminish hepatic tissue injury associated with AHI while decreasing ALT and AST expression levels. Therefore, it is presumed that quercetin and kaempferol may play a protective effect on the liver as important components of THF.

Inflammation is a common pathogenesis in most AHI. LPS activates macrophages and neutrophils to secrete a variety of inflammatory cytokines (TNF-α, IL-6, etc.) to promote the overproduction of inflammatory mediators, which ultimately exacerbates liver injury [16]. This study demonstrated that after administration of THF, liver injury in AHI mice was reduced, the serum levels of inflammatory factors (TNF-α, IL-6, CRP) were decreased, the levels of the anti-inflammatory factor IL-10 increased, and acute inflammatory injury was alleviated. As in this study, THF has been reported to reduce inflammatory factors such as IL-6 and TNF-α, thereby alleviating inflammatory diseases [17]. This suggested that THF may treat AHI by inhibiting the secretion of inflammatory factors and activating anti-inflammatory factors in vivo.

The PPI network analysis also revealed correlation with inflammatory factors. Meanwhile, KEGG analysis showed a link between the PI3K/AKT signaling pathway and THF treatment of AHI. The AKT1 protein, belonging to the AKT signaling pathway family, is crucial in process like cell apoptosis, proliferation, transcription, and various other cellular processes [18]. When AHI occurred, large amounts of cytokines and activation products such as TNF-α were produced [19]. PI3K, as an upstream protein of AKT, can be activated by Toll-like receptors and other pathogen-recognizing receptors, cytokines, and chemokine receptors, such as TNF-α [20]. After PI3K activation, the downstream AKT protein is stimulated and converted to p-AKT. Subsequently, the activated AKT activates the downstream target proteins, regulates the immune response, generates inflammation, and triggers apoptosis [21]. The current study also indicated a reduction in p-AKT and p-PI3K expression and a decrease in inflammatory factor release after THF was given. It was then presumed that THF may have had a protective effect on the liver in this experiment by inhibiting the activation of the PI3K/AKT pathway.

AHI can activate the PI3K/AKT signaling pathway and increase the expression of its downstream related genes [22]. BCL-2 and BAX as apoptotic proteins downstream of the PI3K/AKT pathway are the main regulatory proteins of apoptosis [23], and the level of their expression is directly related to the regulation of cell apoptosis, with increased BCL-2 inhibiting apoptosis and increased BAX promoting apoptosis [24, 25]. All in all, the ratio of BAX/BCL-2 plays an important role in the process of apoptosis [26, 27]. After THF treatment of AHI, the level of apoptosis was inhibited, which led to the improvement of liver viability. THF can increase the expression of BCL-2 protein and decrease the expression of BAX protein in liver tissues, leading to a decrease in the BAX/BCL2 ratio, which reduces apoptosis. TUNEL results also showed that fluorescence positivity was reduced after THF intervention. It proved that THF could reduce the rate of apoptosis in AHI.

## 5. Conclusion

In summary, this study is based on the prediction of the multicomponent, multitarget, and multipathway effects of THF intervention in AHI using network pharmacology. Further animal experimental models have validated that the effect of THF intervention in AHI mice may mainly involve the inhibition of the PI3K/AKT signaling pathway, lowering the BAX/BCL-2 ratio, reduces apoptosis, and ultimately decreased the release of inflammatory factors, which had a protective effect on the liver. The study elucidated the preliminary pharmacological basis and mechanism of action underlying the therapeutic effects of THF in AHI, thereby presenting a promising candidate for clinical treatment. However, the potential molecular mechanism of anti-inflammatory effect requires further exploration. In-depth clarification of the protective effects of THF on AHI would be explored for further research.

## Figures and Tables

**Table 1:** Active ingredients of THF.

	Molecule ID	Molecule name	OB (%)	DL	Number of targets
1	MOL004564	Kaempferide	73.41	0.27	17
2	MOL000004	Procyanidin B1	67.87	0.66	11
3	MOL000492	(+)-catechin	54.83	0.24	10
4	MOL000354	Isorhamnetin	49.6	0.31	31
5	MOL000098	Quercetin	46.43	0.28	74
6	MOL000422	Kaempferol	41.88	0.24	43
7	MOL002322	Isovitexin	31.29	0.72	7

Abbreviations: DL, drug-likeness; OB, oral bioavailability; THF, *Tetrastigma hemsleyanum* Diels et Gilg.

**Table 2:** Molecular docking results.

Active compound	Target	PDB ID	Binding energy (kcal/mol)
Kaempferol	TNF	7jra	−8.07
Quercetin	TNF	7jra	−7.51
Kaempferol	IL-6	1alu	−5.84
Quercetin	IL-6	1alu	−5.88
Kaempferol	TP53	8dc6	−7.01
Quercetin	TP53	8dc6	−5.63

Abbreviations: IL-6, interleukin-6; TNF, tumor necrosis factor.

## Data Availability

All data supporting the findings of this study are available within the paper and its Supporting Information.

## Nomenclature

AHI: Acute hepatic injury
AKT1: Protein kinase B
ALT: Alanine aminotransferase
AST: Aspartate aminotransferase
BAX: BCL2-associated X
BCL-2: B-cell lymphoma-2
BP: Biological process
CC: Cell component
CRP: C-reactive protein
GO: Gene Ontology
IL-10: Interleukin-10
IL-6: Interleukin-6
KEGG: Kyoto Encyclopedia of Genes and Genomes
LPS: Lipopolysaccharide
MF: Molecular function
p-AKT1: Phosphorylated protein kinase B
PI3K: Phosphoinositide 3-kinase
p-PI3K: Phosphorylated phosphoinositide 3-kinase
TCM: Traditional Chinese medicine
TCMSP: Traditional Chinese Medicine Systems Pharmacology
TH: *Tetrastigma hemsleyanum* Diels et Gilg
THF: *Tetrastigma hemsleyanum* Diels et Gilg flavonoids
TNF-α: Tumor necrosis factor alpha.

## Supporting Information

Supporting Information 1 Table S1: The purity of the THF powder.

Supporting Information 2 Data S2: Reagents.
