# Supplementary material for: Flavonoids of Tetrastigma hemsleyanum Diels et Gilg Against Acute Hepatic Injury by Blocking PI3K/AKT Signaling Pathway
Source: Mediators Inflamm. 2025 Jan 7;2025:4302130. doi: 10.1155/mi/4302130 (PMC11732288; doi:10.1155/mi/4302130)
Supplement: Supporting Information 2 — Data S2: Reagents. [file 4302130.f2.docx]

**Flavonoids of *Tetrastigma hemsleyanum* Diels et Gilg against Acute Hepatic Injury by blocking PI3K/AKT signaling way**

1 Reagents

Lipopolysaccharide (LPS, Shanghai, Sigma-Aldrich Company, Batch No.: #0000110441), aspartate aminotransferase (AST, Batch No.: C009-2-1), alanine aminotransferase (ALT, Batch No.: C010-2-1), DAB chromogenic kit (20 × ) ( Batch No.:W026-1-1) were purchased from Nanjing Jiancheng Bioengineering Institute. Improved Citrate Antigen Retrieval Solution (Batch No.: P0083) was purchased from Beyotime Biotechnology. Modified Masson’s Trichrome Stain Kit was purchased from Shanghai yuanye Bio-Technology Co., Ltd. (Batch No.:M08IR209079). Interleukin-10 (IL-10, Batch No.: Cat.#m1037873V), interleukin-6 (IL-6, Batch No.: Cat.#m1063159V), tumor necrosis factor (TNF-α, Batch No.: Cat.#m1002095V), C-reactive protein (CRP, Batch No.: m1002192-C) were obtained from Shanghai Enzyme-linked Biotechnology Co., Ltd. Protein kinase B (AKT1, Batch No.: AF8306), phosphoinositide3-kinase (PI3K, Batch No.: AF6241), phosphorylated phosphoinositide3-kinase (p-PI3K, Batch No.: AF3424), BCL2-Associated X (BAX, Batch No.: 50599-2-Ig) and B-cell lymphoma-2 (BCL-2, Batch No.: 26593-1-AP), HRP-IgG (Batch No.: SA00001-2, Batch No.: SA00001-2) were provided by Affinity Biosciences Co., Ltd. Beta-actin (Batch No.: 66009-I-Ig ) and phosphorylated Protein kinase B (p-AKT1, Batch No.: 28731-1-AP) were provided by Proteintech Group, Inc. The standards of quercetin (PS012150), kaempferol (PS012693) and vitexin (PS010335)were bought from Chendu Push Bio-technology Co., Ltd. (Chendu, China). , nicotifiorin (MUST-16041507) was bought from Chendu MUST Bio-technology Co., Ltd. (Chendu, China).
